# Predictors of clinically meaningful bone mineral density gains with romosozumab: An explainable machine leaning analysis of a real-world cohort

**DOI:** 10.1016/j.bonr.2025.101890

**Published:** 2025-11-26

**Authors:** David Castro Corredor, Luis Ángel Calvo Pascual

**Affiliations:** a Rheumatology Department, General University Hospital of Ciudad Real, Ciudad Real, Spain; b Instituto de Investigación Sanitaria de Castilla-La Mancha (IDISCAM), Spain; c Department of Quantitative Methods, Comillas Pontifical University(ICADE), Madrid, Spain; d Institute for Research in Technology (IIT), Comillas Pontifical University, Madrid, Spain

**Keywords:** Romosozumab, Osteoporosis, Machine learning, Bone mineral density, Predictors, Real-world evidence

## Abstract

**Objective:**

To evaluate the real-world effectiveness of Romosozumab in postmenopausal women with severe osteoporosis and to identify baseline clinical and biochemical predictors of clinically meaningful bone mineral density (BMD) gains (≥10 %, used for exploratory classification) using an explainable machine-learning approach.

**Methods:**

We conducted a retrospective, observational multicentre study across seven hospitals in Castilla-La Mancha, Spain. Postmenopausal women aged ≥50 years who initiated romosozumab between May 2023 and November 2024 for severe osteoporosis or high fracture risk were included. Lumbar-spine, femoral-neck, and total-hip BMD were assessed by dual-energy X-ray absorptiometry (DXA) at baseline and 12 months. Baseline biochemical variables included serum P1NP, CTX, PTH, vitamin D, calcium, phosphate, alkaline phosphatase, and creatinine. Predictors of a ≥ 10 % BMD gain were examined using elastic-net logistic regression combined with SHapley Additive exPlanations (SHAP) for model interpretability.

**Results:**

Fifty-eight women were analysed (mean ± SD age 71.7 ± 10.0 years; BMI 26.1 ± 4.8 kg/m^2^; mean age at menopause 47.3 ± 6.0 years). Mean 12-month BMD increases were + 15,35 % at the lumbar spine, +12,42 % at the femoral neck, and + 8,62 % at the total hip. The proportion achieving a ≥ 10 % gain was 39 %, 38.1 %, and 31.7 %, respectively. SHAP analysis identified consistent predictors of response: lower baseline BMD, higher phosphate levels, and younger age at menopause were associated with greater gains, whereas elevated PTH and alkaline phosphatase predicted a reduced response. Patients who had not received corticosteroids or NSAIDs in the six months prior to treatment initiation, typically for pain or inflammation, also showed greater increases in BMD.

**Conclusions:**

Romosozumab was effective and well-tolerated in routine clinical practice, yielding meaningful and site-specific gains in BMD. Explainable machine-learning analysis identified physiologically coherent and consistent clinical predictors of ≥10 % response.

## Highlights

• Multicentre real-world study of romosozumab effectiveness in postmenopausal osteoporosis.
• Explainable elastic-net and SHAP analyses identified consistent, site-specific predictors of response.
• Younger age at menopause, low PTH, high phosphate, and absence of NSAID or corticosteroid use in the preceding months predicted ≥10 % BMD gain.
• Findings highlight the importance of baseline skeletal status and treatment context for individualized anabolic therapy.

## 1 Introduction

Osteoporosis is a chronic skeletal disorder characterized by decreased bone mass and deterioration of bone microarchitecture, culminating in increased bone fragility and fracture risk. Loss of estrogen following menopause is one of the most potent drivers of accelerated bone resorption (Riggs et al., 2002; Compston, 2001). Early menopause, therefore, confers a disproportionately high lifetime risk of osteoporosis and fragility fractures. European guidance from IOF/ESCEO indicates that the lifetime probability of sustaining at least one major osteoporotic fracture in women living in Western Europe is 40 % or higher (Kanis et al., 2019).

Romosozumab is a humanised monoclonal antibody that targets sclerostin, an osteocyte-derived inhibitor of the Wnt signalling pathway. By simultaneously stimulating bone formation and suppressing bone resorption, romosozumab promotes rapid gains in bone mineral density (BMD). In pivotal clinical trials, FRAME (Cosman et al., 2016) and ARCH (Saag et al., 2017), 12-month romosozumab treatment increased lumbar-spine BMD by 13.3 % and 13.7 %, respectively, and significantly reduced the risk of new vertebral fractures compared with placebo or alendronate. However, neither trial demonstrated a statistically significant reduction in non-vertebral fractures at 12 months. In ARCH, reductions in non-vertebral and hip fractures became significant at the time of the primary event-driven analysis (non-vertebral *p* = 0.04; hip *p* = 0.02), while in FRAME, the lack of significance at 12 months was partly attributed to the low background fracture incidence in some geographic regions. These findings underscore the potent anabolic effect of Romosozumab on BMD and vertebral fracture risk, while highlighting the importance of confirming the effectiveness and safety of romosozumab outside the constraints of clinical trials and of identifying which patients derive the greatest benefit (Hattori and Kanayama, 2024; Mineta et al., 2024).

Machine-learning (ML) algorithms can integrate a large number of clinical and biochemical variables simultaneously and are capable of capturing complex, non-linear relationships that traditional regression models may fail to detect. In the context of osteoporosis research, ML has shown promise for identifying patient subgroups with differential responses to anabolic or antiresorptive therapies (Chen et al., 2021; Li et al., 2023). Combining ML with explainability frameworks, such as SHapley Additive exPlanations (Lundberg and Lee, 2017), allows visualisation of the relative importance and direction of individual predictors, thus improving interpretability and clinical transparency. In this study, we examined bone mineral density (BMD) responses to romosozumab in postmenopausal women treated in routine practice. We developed an *explainable* ML approach based on elastic-net logistic regression with SHAP analysis to identify baseline predictors of clinically meaningful increases in BMD at the lumbar spine, femoral neck, and total hip defined by a ≥ 10 % increase used for exploratory classification of high responders We compared its performance with conventional L2-regularized logistic regression models to assess the added value of the ML framework.

## 2 Materials and methods

### 2.1 Study design and participants

This retrospective, multicentre observational cohort study was conducted across seven public hospitals in Castilla-La Mancha, Spain. Consecutive postmenopausal women aged ≥50 years who initiated romosozumab between May 2023 and November 2024 were identified from pharmacy and clinic records. Recruitment began in May 2023, coinciding with the consistent regional availability of romosozumab and the implementation of standardized data collection procedures.

Romosozumab was prescribed according to national clinical guidelines for severe osteoporosis or high fracture risk. Eligible patients had a T-score ≤ −2.5 at the lumbar spine, femoral neck, or total hip and/or a documented history of fragility fracture. In accordance with prescribing guidance, romosozumab is contraindicated in patients with recent myocardial infarction, stroke, or untreated hypocalcaemia; therefore, such individuals were not prescribed the drug and were not part of the study cohort. All participants included met the safety criteria for treatment initiation.

Information on previous osteoporosis treatments (e.g., bisphosphonates and denosumab) and romosozumab exposure (dose and duration) was extracted from medical records. Among the 58 women included in the analysis, 33 (57 %) had received prior antiresorptive therapy, including bisphosphonates (*n* = 26) and denosumab (*n* = 7), with a median treatment duration of 4.2 years (interquartile range [IQR] 2.8–6.5) and a median washout period of 8.5 months (IQR 5–14) before starting romosozumab. A small number had previously used hormone replacement therapy (HRT; *n* = 4) or selective estrogen receptor modulators (SERMs; *n* = 3), all discontinued more than five years before baseline. No participants had been treated with teriparatide or abaloparatide.

The final cohort comprised 58 treated women, some of whom lacked evaluable 12-month BMD data at all sites. Consequently, site-specific analyses were restricted to patients with both baseline and 12-month BMD data available for the corresponding skeletal region (41 for the lumbar spine, 42 for the femoral neck, and 41 for the total hip).

DXA scans of the lumbar spine (L1–L4), femoral neck, and total hip were performed at baseline and 12 months. Different DXA scanners were used across centres, but all devices were calibrated according to manufacturer specifications and International Society for Clinical Densitometry (ISCD) guidelines. When necessary, cross-calibration was performed using phantom scans to harmonise measurements across sites. Fasting morning blood samples were collected at baseline, six, and twelve months to determine serum P1NP, CTX, PTH, 25-hydroxyvitamin D, calcium, phosphorus, alkaline phosphatase, and creatinine. Bone turnover markers were analysed locally using standardized, quality-controlled assays. Although trabecular bone score (TBS) can be derived from lumbar spine DXA images and provides complementary information on trabecular microarchitecture, TBS data were not available across all participating centres.

Fractures that occurred during treatment were recorded over the follow-up period from treatment initiation to 12 months through review of clinical records and radiographic reports and were classified as vertebral or non-vertebral. Adverse events, including injection-site reactions, hypocalcaemia, and cardiovascular events, were documented throughout the follow-up period. If a patient developed hypocalcaemia or a cardiovascular event, romosozumab was temporarily or permanently discontinued at the discretion of the treating physician, in accordance with safety recommendations.

The variable line of therapy was defined as the sequence of osteoporosis therapies, indicating whether romosozumab was administered as first, second, or third-line treatment. Concomitant medications were recorded at baseline and defined as pharmacologic agents with potential effects on bone metabolism that had been used within the six months preceding romosozumab initiation, irrespective of indication or treatment duration. These included non-steroidal anti-inflammatory drugs (NSAIDs), systemic corticosteroids, antacids, thyroid-altering medications, and antiepileptic agents.

#### 2.1.1 Methodological Framework

Descriptive statistical analyses were conducted to characterize the overall study population. For numerical variables, we report measures of central tendency and dispersion (mean, median, standard deviation, and quartiles), together with the proportion of missing data. These results, stratified at baseline, 6 months, and 12 months, are presented in Table 1. Categorical variables were summarized by absolute and relative frequencies, with complete distributions available in the supplementary material (*Descriptive.xlsx*).

From this point onward, all subsequent analyses were performed using full cases only—defined as patients with both baseline and 12-month BMD data for the corresponding skeletal site (lumbar spine, femoral neck, or total hip). As all subsequent analyses relied on directly observed BMD outcomes, a dedicated descriptive analysis was performed for the full-case cohort to characterize the exact population included in the inferential and modeling phases. BMD was expressed as either T-score or g/cm^2^, which are equivalent for longitudinal assessment and were used consistently within each site. Descriptive characteristics of the full-case subsets are provided in Descriptive_FullCases.xlsx.

We characterized the empirical distribution of bone mineral density (BMD) gains by computing the 12-month percentage change from baseline for lumbar spine, femoral neck, and total hip, defined as

$$\mathrm{Improvement}\;\left(\mathrm{BDM}\right)=\frac{\mathrm{BM}D_{12\;\mathrm{Months}}-\mathrm{BD}M_{\mathrm{Baseline}}}{\mathrm{BD}M_{\mathrm{Baseline}}}$$

**Table 1:** Baseline characteristics of the study population (*n* = 58).

Variable	Missing n (%)	Mean	25th Percentile	Median	75th Percentile	Standard Deviation
Age (years)	0 (0.0 %)	71,74	66	73	78	10,31
BMI (kg/m^2^)	0 (0.0 %)	26,11	22,32	24,85	29,05	4,82
Age at menopause (years)	1 (1.7 %)	47,26	45	48	51	5,82
Days on romosozumab	0 (0.0 %)	365,47	364	365	369,75	51,2
Lumbar spine BMD T-score (baseline)	2 (3.4 %)	−2,97	−3,8	−3,2	−2,48	1,37
Lumbar spine BMD (g/cm^2^) baseline	2 (3.4 %)	0,78	0,51	0,67	0,85	0,13
Femoral neck BMD T-score (baseline)	1 (1.7 %)	−2,61	−3,1	−2,6	−1,9	0,91
Femoral neck BMD (g/cm^2^) baseline	1 (1.7 %)	0,6	0,56	0,67	0,74	0,13
Total hip BMD T-score (baseline)	0 (0.0 %)	−2,55	−3,19	−2,55	−1,8	0,94
Total hip BMD (g/cm^2^) baseline	0 (0.0 %)	0,66	0,56	0,67	0,74	0,13
FRAX hip risk (baseline, %)	10 (17.2 %)	10,75	3	6,65	12,5	12,85
Lumbar spine BMD T-score (12 m)	16 (27.6 %)	−2,02	−3,18	−2,3	−1,42	1,66
Lumbar spine BMD (g/cm^2^) 12 m	16 (27.6 %)	0,9	0,77	0,85	1	0,18
Femoral neck BMD T-score (12 m)	16 (27.6 %)	−2,3	−2,88	−2,35	−1,7	0,83
Femoral neck BMD (g/cm^2^) 12 m	16 (27.6 %)	0,66	0,59	0,65	0,74	0,1
Total hip BMD T-score (12 m)	17 (29.3 %)	−2,24	−3	−2,2	−1,6	0,94
Total hip BMD (g/cm^2^) 12 m	17 (29.3 %)	0,71	0,62	0,72	0,8	0,11
FRAX hip risk (12 m, %)	24 (41.4 %)	7,35	3,35	5,55	9,82	5,18
Creatinine (baseline)	0 (0.0 %)	0,76	0,6	0,7	0,87	0,2
Corrected calcium (baseline)	0 (0.0 %)	9,42	9,12	9,4	9,7	0,44
Phosphate (baseline)	3 (5.2 %)	3,54	3,2	3,5	3,8	0,66
Alkaline phosphatase (baseline)	5 (8.6 %)	82,38	60	73	93	35,12
PTH (baseline)	0 (0.0 %)	68,52	40,17	60,15	91,15	33,23
25-OH Vitamin D (baseline)	1 (1.7 %)	35,73	25	32	44,7	15,48
P1NP (baseline)	43 (74.1 %)	46,2	24,5	36,2	63,6	29,96
CTX (baseline)	30 (51.7 %)	0,4	0,16	0,29	0,6	0,33
Creatinine (6 m)	17 (29.3 %)	0,76	0,67	0,72	0,86	0,16
Corrected calcium (6 m)	17 (29.3 %)	9,43	9,1	9,4	9,7	0,36
Phosphate (6 m)	18 (31.0 %)	3,52	3,18	3,45	4	0,62
Alkaline phosphatase (6 m)	20 (34.5 %)	98,3	75,5	87	108	36,55
PTH (6 m)	18 (31.0 %)	74,19	41,58	63,3	88,85	45,09
25-OH Vitamin D (6 m)	18 (31.0 %)	37,82	31,5	38	43,6	11,79
P1NP (6 m)	32 (55.2 %)	79,02	45,8	58,4	101,42	63,84
CTX (6 m)	25 (43.1 %)	0,47	0,16	0,37	0,52	0,5
Creatinine (12 m)	15 (25.9 %)	0,79	0,66	0,75	0,9	0,19
Corrected calcium (12 m)	16 (27.6 %)	9,48	9,12	9,4	9,9	0,48
Phosphate (12 m)	17 (29.3 %)	3,38	3	3,3	3,7	0,8
Alkaline phosphatase (12 m)	17 (29.3 %)	87,76	68	74	101	31,45
PTH (12 m)	17 (29.3 %)	77,43	48	63,7	95,3	43,32
25-OH Vitamin D (12 m)	19 (32.8 %)	35,68	25,9	35,7	43	19,62
P1NP (12 m)	34 (58.6 %)	53,12	26,5	37,75	61,12	40,45
CTX (12 m)	31 (53.4 %)	0,67	0,17	0,29	0,64	0,96

**Fig. 1:**
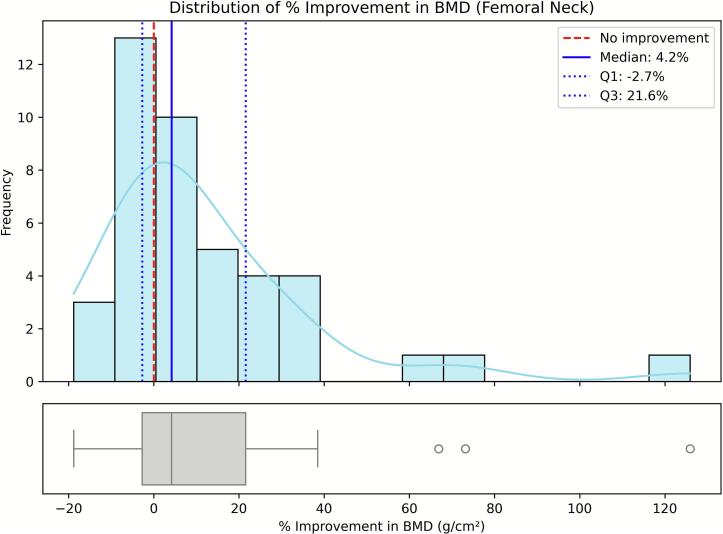
Distribution of the 12-month percentage change in bone mineral density (BMD) at the femoral neck (*N* = 42). Histograms show the frequency of percentage change among patients with both baseline and 12-month BMD measurements for this site (complete cases). The red dashed line indicates no improvement (0 %), the blue solid line marks the sample median, and the blue dotted lines represent the interquartile range (Q1–Q3).

**Fig. 2:**
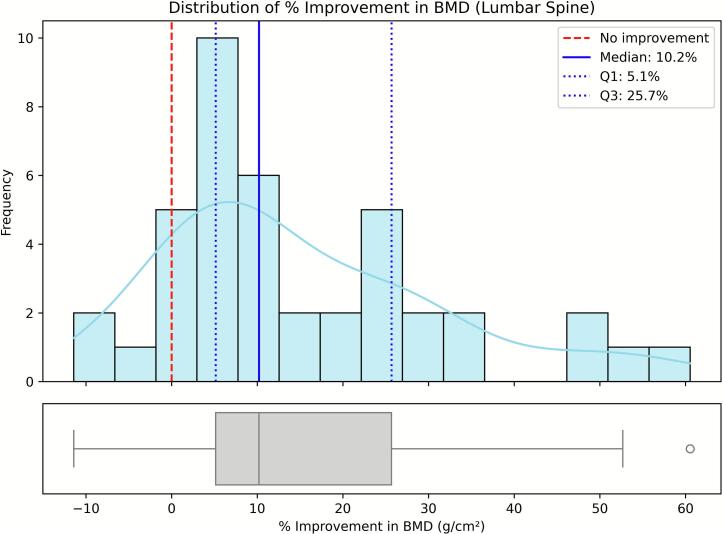
Distribution of the 12-month percentage change in bone mineral density (BMD) at the lumbar spine (*N* = 41). Histograms show the frequency of percentage change among patients with both baseline and 12-month BMD measurements for this site (complete cases). The red dashed line indicates no improvement (0 %), the blue solid line marks the sample median, and the blue dotted lines represent the interquartile range (Q1–Q3).

**Fig. 3:**
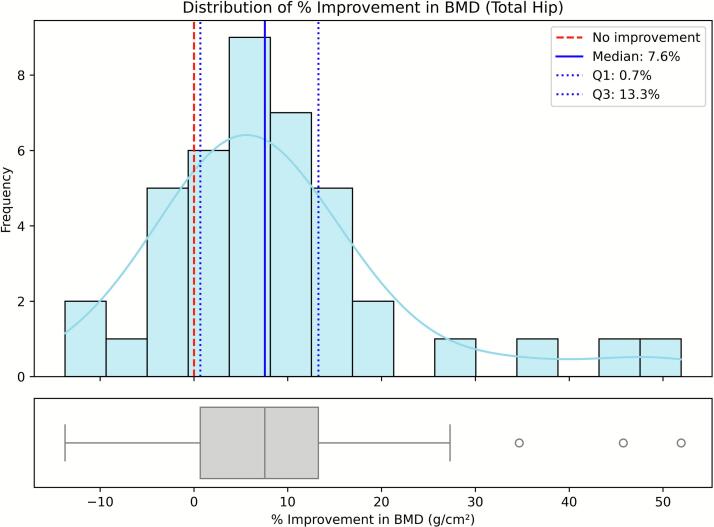
Distribution of the 12-month percentage change in bone mineral density (BMD) at the total hip (N = 41). Histograms show the frequency of percentage change among patients with both baseline and 12-month BMD measurements for this site (complete cases). The red dashed line indicates no improvement (0 %), the blue solid line marks the sample median, and the blue dotted lines represent the interquartile range (Q1–Q3).

For each of the three skeletal sites, lumbar spine, femoral neck, and total hip, we generated univariate histograms (15 bins) combined with aligned boxplots on the same scale. The histograms include reference markers at 0 % (no improvement), the sample median, and the first and third quartiles, while the boxplots emphasize the interquartile range and potential outliers. The resulting distributions are shown in Fig. 1, Fig. 2, Fig. 3, corresponding to lumbar spine, femoral neck, and total hip, respectively.

Given the relatively small dataset (40 patients for the lumbar spine, and 39 each for the femoral neck and total hip), and the presence of missing data in some variables we applied a multiple imputation strategy to reduce bias and enhance the robustness of subsequent analyses. Numerical variables were imputed using an iterative regression-based approach with posterior sampling, while categorical variables were imputed using multivariate imputation by chained equations (MICE) with logistic or multinomial regression models, which better preserve associations among variables than simple mode imputation. Following established recommendations that the number of imputations should be at least equal to the percentage of incomplete cases (White et al., 2011), we generated 40 multiply imputed datasets.

We evaluated feature contributions using SHAP values computed on elastic-net logistic regression models trained to predict binary responder outcomes for lumbar spine, femoral neck, and total hip BMD. Responders were defined as patients achieving a ≥ 10 % improvement at 12 months compared with baseline. The 10 % threshold was selected because it corresponded approximately to the upper quartile of the observed BMD gain distributions across skeletal sites. This cut-off allowed us to focus on patients exhibiting the most pronounced anabolic responses, while maintaining adequate group balance for modeling. Although smaller increases in BMD (typically 3–6 %) have been associated with fracture-risk reduction in large clinical trials, the purpose of this classification was to distinguish high responders for exploratory analysis rather than to define a clinical treatment target (Fig. 1, Fig. 2, Fig. 3).

**Fig. 4:**
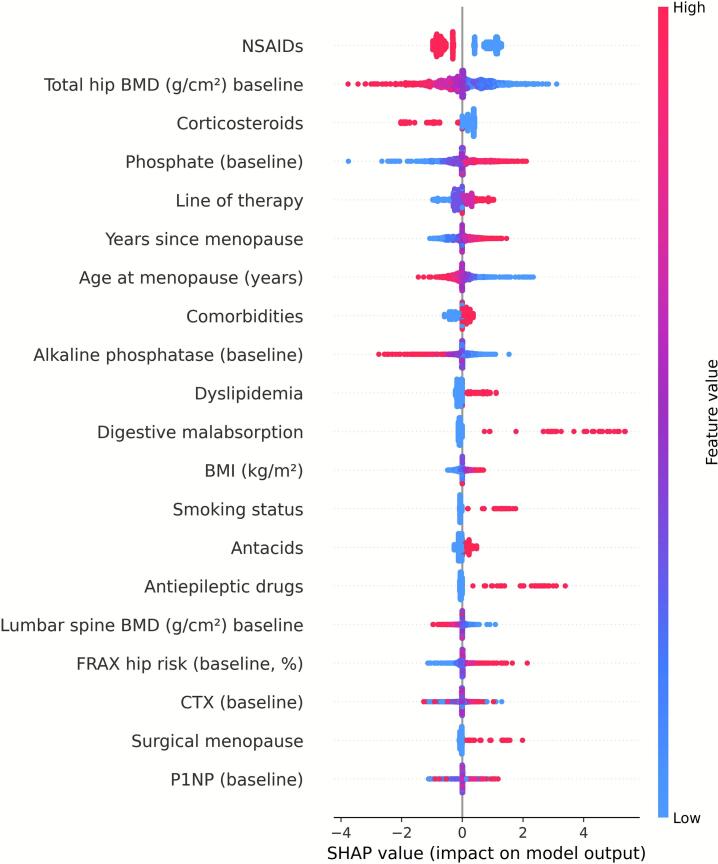
SHAP beeswarm plot of baseline predictors for ≥10 % femoral neck BMD gain. The analysis includes only patients with both baseline and 12-month BMD measurements for this site (complete cases). Variables are ranked from top to bottom by mean absolute SHAP value, with each point representing an individual patient. Red indicates higher feature values, blue indicates lower values.

**Fig. 5:**
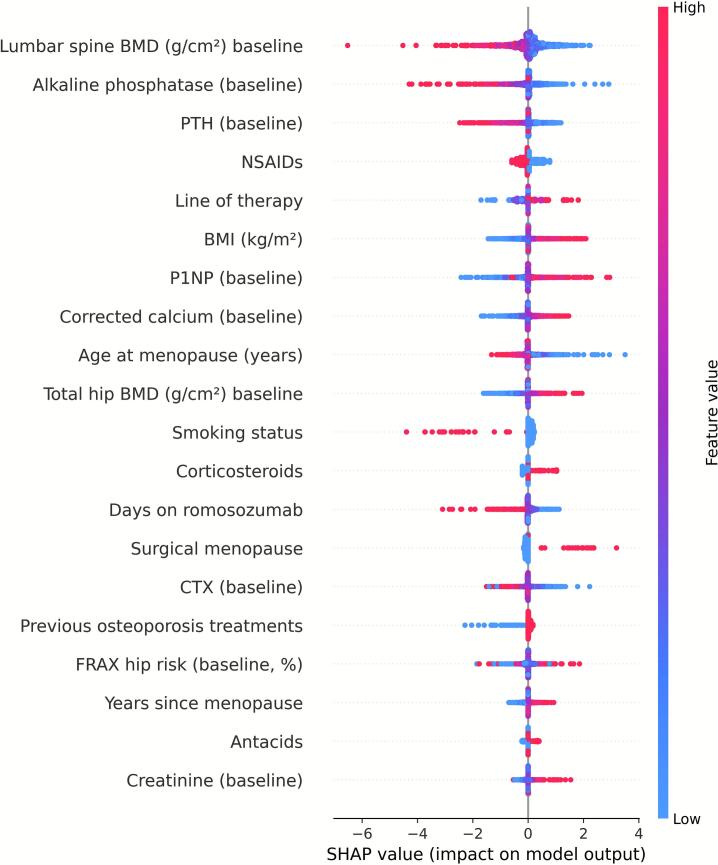
SHAP beeswarm plot of baseline predictors for ≥10 % lumbar spine BMD gain. The analysis includes only patients with both baseline and 12-month BMD measurements for this site (complete cases). Variables are ranked from top to bottom by mean absolute SHAP value, with each point representing an individual patient. Red indicates higher feature values, blue indicates lower values.

**Fig. 6:**
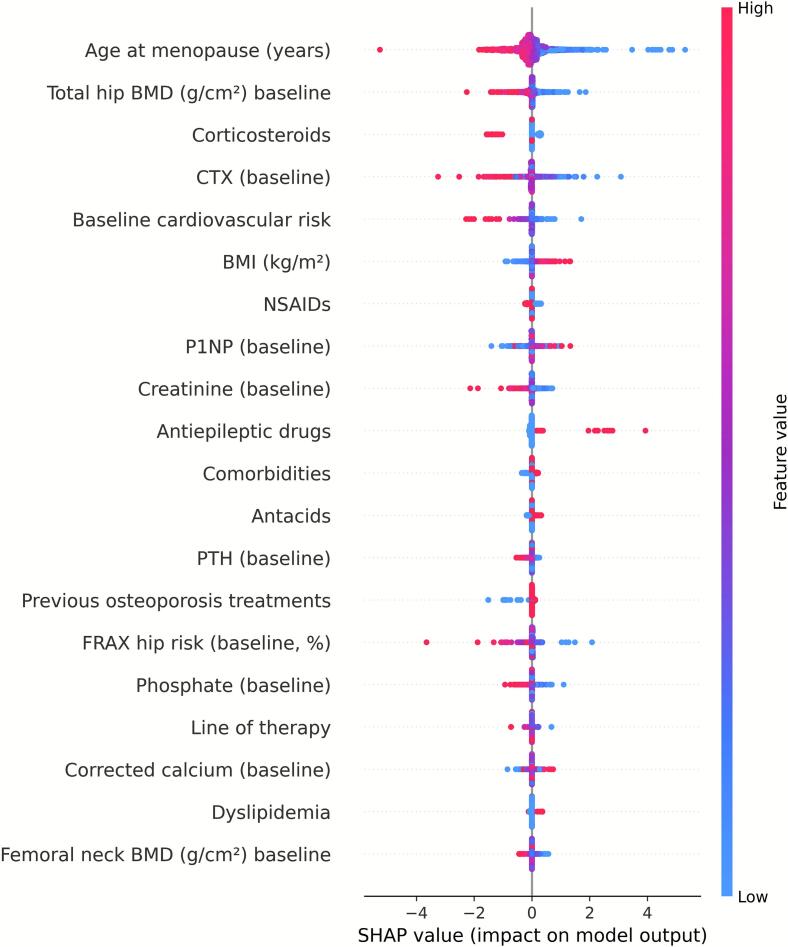
SHAP beeswarm plot of baseline predictors for ≥10 % total hip BMD gain. The analysis includes only patients with both baseline and 12-month BMD measurements for this site (complete cases). Variables are ranked from top to bottom by mean absolute SHAP value, with each point representing an individual patient. Red indicates higher feature values, blue indicates lower values.

Before modeling, we excluded follow-up or derived variables to avoid information leakage (i.e., inadvertent use of post-baseline information that could artificially improve model performance) and applied a standardized preprocessing pipeline (z-scaling for numerical variables, one-hot encoding with rare-level collapsing for categorical variables). Categorical predictors with sparse categories collapsed into broader, clinically coherent groups to ensure stable estimation. Specifically, categories represented by fewer than three patients were merged with the nearest related group. Elastic-net logistic regression models were optimized by stratified cross-validation and refitted across 40 multiply imputed datasets for each skeletal site. SHAP values were then computed, aligned, and aggregated to provide consistent feature attributions. SHAP values were computed for each of the 40 multiply imputed datasets and aggregated to yield mean absolute SHAP values, thereby incorporating imputation variability and enhancing stability of feature importance estimates. Results are summarized as mean absolute SHAP values, visualized in beeswarm plots (Fig. 4, Fig. 5, Fig. 6 for lumbar spine, femoral neck, and total hip) and detailed in the supplementary Excel file (*shap_results.xlsx*). This approach propagates imputation variance into the explanations and stabilizes estimation under high-dimensional encoding (Zou and Hastie, 2005).

As a baseline comparator, we fitted L2-regularized logistic regression models (solver = liblinear, class-weight = balanced) for lumbar spine, femoral neck, and total hip responders (≥10 % at 12 months), applying the same pre-treatment predictors and preprocessing as in the SHAP pipeline. Models were refitted across M = 40 multiply imputed datasets; coefficients were aggregated across imputations (mean β and empirical 95 % CIs from the imputation distribution), and performance (ROC-AUC, PR-AUC, Brier, calibration) was estimated via stratified 5-fold cross-validation within each imputation and summarized across imputations. The full set of regression coefficients and performance metrics is provided in the supplementary material (*logit_results_L2.xlsx*).

All analyses were performed in Python 3.10 using scikit-learn v1.4.2 and shap v0.45.1. The elastic-net logistic regression models were implemented via LogisticRegression (penalty = “elasticnet”, solver = “saga”), with hyperparameters tuned through stratified 5-fold cross-validation within each multiply imputed dataset. The regularization strength (C) was searched over [0.05–1.0] and the mixing parameter (l1_ratio) over [0.1–0.9]. SHAP values were computed using shap.Explainer with default settings. For linear estimators, this function automatically applies the LinearExplainer, yielding exact additive attributions on the log-odds scale using the preprocessed design matrix as background. SHAP values from the 40 imputations were column-aligned, stacked, and averaged to obtain global feature importance.

#### 2.1.2 Ethics

The study protocol was reviewed and approved by the central research ethics committee of Ciudad Real (study ID: 12/2023) and by the local committees of participating centres. Written informed consent was obtained from all participants. The study adhered to the principles of the Declaration of Helsinki.

## 3 Results

A total of 58 postmenopausal women were included. Mean (± SD) age was 71.7 ± 10.0 years, mean BMI was 26.1 ± 4.8 kg/m^2^, and the mean age at menopause was 47.3 ± 6.0 years. Ten percent of participants had undergone surgical menopause. The mean cardiovascular risk score (REGICOR) was 3.1 ± 3.2. The majority of patients (89.7 %) had received previous osteoporosis therapy (mainly bisphosphonates or denosumab). Table 1 and the supplementary material descriptive.xlsx report the descriptive characteristics of the study cohort, including demographic, clinical, and biochemical variables at baseline, 6 months, and 12 months. During follow-up, two women (3.4 %) sustained vertebral fractures. No major cardiovascular events occurred. Six participants (10.3 %) reported minor adverse effects such as headache or injection-site irritation. Romosozumab was discontinued early in two patients due to adverse events.

Mean percentage change in BMD at 12 months, calculated for complete cases at each skeletal site, was 15.35 % (SD 16.57 %) at the lumbar spine (*n* = 41), 12.42 % (SD 26.29 %) at the femoral neck (*n* = 42), and 8.62 % (SD 13.23 %) at the total hip (n = 41). The proportion of participants achieving a ≥ 10 % BMD increase was 39.0 % at the lumbar spine, 38.1 % at the femoral neck, and 31.7 % at the total hip. Most patients maintained or improved BMD; deterioration exceeding 10 % occurred in 5 % at the lumbar spine and 7 % at the femoral neck, while no marked decline (>10 %) was observed at the total hip. The distributions of percentage increases in BMD were markedly skewed and deviated from normality across all skeletal sites (Fig. 1, Fig. 2, Fig. 3). This non-Gaussian distribution justified the use of nonparametric summaries (median, quartiles) and the definition of a binary responder outcome (≥10 % 12-month gain) to identify patients with the most pronounced improvements.

Fig. 4, Fig. 5, Fig. 6 display SHAP summary plots for the models predicting ≥10 % BMD gain. Each point represents one participant; the horizontal axis indicates the effect of the variable on the model's output, and colour reflects the variable's magnitude (red = high, blue = low). Features are ordered by their mean contribution to prediction probability.

For the femoral neck, the main predictors of BMD gain (≥10 % at 12 months) identified by SHAP were NSAIDs (mean |SHAP| = 0.80), baseline total hip BMD (g/cm^2^) (0.66), corticoids (mean |SHAP| = 0.39), baseline phosphate (0.37), Line of therapy (mean |SHAP| = 0.27) and years since menopause (mean |SHAP| = 0.27), (Fig. 4 and Supplementary material, shap_femoral.xlsx).

For the lumbar spine, the strongest SHAP predictors of ≥10 % BMD gain at 12 months were baseline lumbar spine BMD (mean |SHAP| = 0.41), baseline alkaline phosphatase (0.28), baseline PTH (mean |SHAP| = 0.26) and line of therapy (mean |SHAP| = 0.23). Age at menopause had a slightly smaller influence (mean |SHAP| = 0.17). (Fig. 5 and Supplementary material, shap_lumbar.xlsx).

For the total hip, age at menopause was the main SHAP predictor of a ≥ 10 % BMD gain (mean |SHAP| = 0.3440), followed, at roughly one-third of its influence, by baseline total hip BMD and corticosteroid use (both 0.12).(Fig. 6 and Supplementary material, shap_totalhip.xlsx).

Across skeletal regions, the elastic-net logistic models demonstrated solid discriminative performance and acceptable calibration. The femoral-neck model achieved the highest discrimination (ROC-AUC = 0.98, PR-AUC = 0.97), while the total-hip and lumbar-spine models also showed good performance (ROC-AUC = 0.90 and 0.86, respectively). Calibration was adequate in all cases (Brier scores between 0.06 and 0.18), with balanced sensitivity and specificity near 0.9. These results indicate that model performance was consistent across sites, supporting the reliability of SHAP-derived feature attributions. Full cross-validated performance metrics and site-specific SHAP summaries are provided in the Supplementary Material (shap_femoral.xlsx, shap_totalhip.xlsx, shap_lumbar.xlsx).

## 4 Discussion

When compared with the conventional logistic regression model, the femoral-neck analysis yielded results consistent with those obtained from the SHAP approach. The largest regression coefficients were observed for NSAID use (β = −0.94, 95 % CI −1.05 to −0.81), corticosteroid use (β = −0.83, 95 % CI −0.91 to −0.69), baseline total hip BMD (β = −0.57, 95 % CI −0.63 to −0.50), baseline phosphate levels (β = +0.80, 95 % CI 0.68 to 0.90), and age at menopause (β = −0.54, 95 % CI −0.67 to −0.40)(Fig. 4 and Supplementary materials: logit_results_l2.xlsx).

In the lumbar spine model, the main predictors identified by the SHAP analysis were largely consistent with those obtained from the logistic regression model. Both approaches highlighted baseline lumbar spine BMD (β = −0.85, 95 % CI −1.20 to −0.47), baseline alkaline phosphatase (β = −0.89, 95 % CI −1.11 to −0.64), and baseline PTH (β = −0.74, 95 % CI −1.00 to −0.44) as the most influential variables. The logistic regression model assigned slightly less importance to the line of therapy (β = +0.54, 95 % CI 0.11 to 0.94) and a somewhat greater weight to age at menopause (β = −0.68, 95 % CI −1.08 to −0.07), reflecting a similar overall pattern of associations between both analytical approaches (Fig. 5 and Supplementary materials: logit_results_l2.xlsx).

In the total hip model, both the SHAP analysis and the logistic regression identified age at menopause (β = −1.50, 95 % CI −1.70 to −1.24) and corticosteroid use (β = −0.78, 95 % CI −0.91 to −0.59) as the most influential predictors of ≥10 % BMD gain. However, the third most relevant variable differed between approaches: in the logistic regression model, line of therapy (β = −0.59, 95 % CI −0.79 to −0.38) or baseline phosphate (β = −0.36, 95 % CI −0.60 to −0.18) ranked higher than baseline total hip BMD (β = −0.34, 95 % CI −0.58 to −0.10). This minor discrepancy could be explained by differences in how each method captures nonlinear relationships and complex interactions among predictors (Fig. 6 and Supplementary materials: logit_results_l2.xlsx).

Our real-world results (mean + 15.35 % spine; +12.42 % femoral neck; +8.62 % total hip at 12 months) fall within the efficacy range reported in pivotal trials such as FRAME and ARCH, where romosozumab produced ∼13–14 % lumbar-spine gains and significantly reduced vertebral fractures at 12 months (Cosman et al., 2016; Saag et al., 2017). The more modest hip responses we observed mirror known site-specific differences and patient heterogeneity outside RCTs.

A Swiss multicentre cohort similarly demonstrated that prior antiresorptive exposure attenuated romosozumab gains at both spine and hip (Everts-Graber et al., 2024), consistent with our inverse association between baseline bone status and relative BMD change. Likewise, Korean real-world data indicate potential synergy between romosozumab and menopausal hormone therapy (MHT), paralleling our strong “concomitant-medication” signal (Park et al., 2025).

The inverse relationship between baseline BMD and relative gains is expected for an anabolic-antiresorptive agent and reflects the greater “remodeling space” in severely osteoporotic bone. Comparable findings were reported in post-hoc analyses of FRAME and in real-world series (Cosman et al., 2018).

At the total hip, age at menopause emerged as a dominant SHAP predictor, whereas it was less influential for the lumbar spine or femoral neck. This site-specificity is biologically plausible: hip bone integrates long-term systemic hormonal effects, while spine response depends more on local turnover dynamics and recent pharmacotherapy. Previous longitudinal studies have shown that later menopause is associated with higher hip BMD and slower postmenopausal bone loss, whereas spine BMD tends to reflect shorter-term remodeling changes (Sowers et al., 2003; Slemenda et al., 1996). These findings support a nuanced, site-dependent relationship between menopausal age and skeletal adaptation.

Baseline phosphate emerged as a positive predictor in both the lumbar and femoral models. Adequate phosphate may enhance mineralization and support the anabolic effect of romosozumab. Elevated baseline PTH, which was influential in the lumbar model, predicted a poorer spine response, underscoring the importance of ensuring sufficient vitamin D and calcium before initiating therapy—not only to correct secondary hyperparathyroidism but also to optimize bone metabolism and the anabolic response (Reid et al., 1995; Drake et al., 2017).

Absence of corticosteroids or NSAIDs in the six months preceding romosozumab initiation, often prescribed for pain or inflammatory conditions, was associated with greater increases in BMD. This finding likely reflects a subgroup with lower inflammatory burden and more stable musculoskeletal status at treatment onset, rather than a direct pharmacological effect on bone remodeling.

Our dataset was relatively small (*n* = 58), which limited statistical power and generalizability. When restricting the analysis to complete cases, the effective sample size decreased to 39–40 participants. Missing data (∼40 % for some covariates) were handled using multiple imputation by chained equations (m = 40), consistent with best-practice recommendations (White et al., 2011). Elastic-net logistic regression, optimized via cross-validation, mitigated overfitting and accommodated correlated predictors, while SHAP values enhanced transparency and interpretability (Zou and Hastie, 2005). However, given the limited sample size, individual SHAP estimates may still be unstable despite aggregation across imputations, and their interpretation should be considered exploratory. Model discrimination was strong (AUC = 0.83–0.91) and calibration acceptable (Brier = 0.14–0.17), supporting internal validity despite sample constraints. Despite good internal performance and calibration, our model has not yet undergone external validation. Future multicentre or registry-based studies with larger samples will be necessary to confirm its generalizability and to test SHAP stability in independent cohorts.

To further assess the robustness and clinical interpretability of our models, we repeated the SHAP analysis using an inverse outcome, predicting cases with BMD loss instead of ≥10 % gain. (Supplementary materials: worsening.zip). The rationale for this complementary analysis was twofold: first, to determine whether the predictors of improvement mirrored those associated with deterioration, and second, to identify clinical or treatment-related factors linked to suboptimal responses. For the total hip, the most influential predictors of bone loss were baseline 25-OH vitamin D (mean |SHAP| = 0.79), comorbidities (0.67), hypertension (0.64), and NSAID use (0.58), followed by baseline PTH (0.45). For the femoral neck, comorbidities (0.60), baseline femoral-neck BMD (0.50), baseline lumbar-spine BMD (0.42), prior fractures (0.37), baseline 25-OH vitamin D (0.28), BMI (0.26), and NSAIDs use (0.18) contributed most strongly to the model. These findings indicate that poorer bone outcomes were associated with a less favorable metabolic profile, characterized by lower vitamin D levels, greater comorbidity burden, and vascular or inflammatory conditions, as well as with higher baseline BMD, consistent with a ceiling effect. The recurrent influence of NSAID exposure across sites may reflect a transient improvement in bone turnover regulation among patients receiving closer clinical follow-up during the early treatment phase.

In summary, our findings highlight that the response to romosozumab is largely determined by the patient's baseline skeletal and metabolic profile. Lower baseline BMD and adequate phosphate levels favored greater gains, whereas higher PTH and alkaline phosphatase were associated with a blunted response, underscoring the need to optimize bone turnover before initiating therapy. Age at menopause emerged as a strong, site-dependent determinant, particularly at the hip, consistent with the cumulative impact of long-term estrogen deficiency on cortical bone remodeling. Moreover, the absence of corticosteroid or NSAID use in the six months preceding treatment initiation was associated with greater early BMD improvements, which likely reflects a subgroup with lower inflammatory burden and more stable musculoskeletal status at treatment onset rather than a direct pharmacologic effect. Overall, these results suggest that individualized management, integrating hormonal, metabolic, and pharmacologic factors, may enhance the efficacy and safety of romosozumab in real-world settings.

## 5 Conclusions

Romosozumab was an effective and well-tolerated anabolic therapy for severe postmenopausal osteoporosis in routine clinical practice. Clinically meaningful increases in BMD were achieved across skeletal sites, with the greatest improvements at the lumbar spine and consistent benefits at the femoral neck and total hip. The integration of explainable machine learning with logistic regression revealed physiologically coherent predictors of response. Greater increases in BMD, defined using an exploratory ≥10 % threshold, were associated with lower baseline bone density and adequate phosphate levels, whereas elevated PTH and alkaline phosphatase were linked to a reduced response. Age at menopause was a key determinant at the total hip and remained relevant at other sites, reflecting the cumulative hormonal influence on bone remodeling. Patients who had not received corticosteroids or NSAIDs in the months preceding treatment initiation experienced greater early BMD increases, likely reflecting lower inflammatory burden and more stable musculoskeletal status rather than a direct pharmacologic effect. Prospective multicentre studies with larger samples are needed to validate these predictors and to assess whether similar response patterns persist when applying smaller, clinically meaningful BMD thresholds associated with fracture-risk reduction.

The following are the supplementary data related to this article.

Supplementary material 1 descriptive.xlsx. A comprehensive overview of both continuous and categorical data. Supplementary material 1

Supplementary material 2 descriptive_FullCases.xlsx. Contains detailed descriptive statistics for the *full-case* analytic subsets, defined as patients with both baseline and 12-month BMD measurements for the corresponding skeletal site (lumbar spine, femoral neck, or total hip). Supplementary material 2

Supplementary material 3 shap_femoral.xlsx. Contains detailed outputs of the SHAP analysis for the femoral-neck model across 40 multiply imputed datasets, including mean absolute SHAP importance, and aggregated performance metrics. Supplementary material 3

Supplementary material 4 shap_totalhip.xlsx Contains detailed outputs of the SHAP analysis for the total hip model across 40 multiply imputed datasets, including mean absolute SHAP importance, and aggregated performance metrics. Supplementary material 4

Supplementary material 5 shap_lumbar.xlsx Contains detailed outputs of the SHAP analysis for the lumbar spine model across 40 multiply imputed datasets, including mean absolute SHAP importance, and aggregated performance metrics. Supplementary material 5

Supplementary material 6 logit_results_l2.xlsx. Results of the baseline L2-regularized logistic regression analyses performed across multiply imputed datasets (M = 40). Supplementary material 6

Supplementary material 7 worsening.zip contains SHAP plots and Excel metrics summarizing model results for patients showing no improvement (≤ 0 % change in BMD after one year of treatment) at the lumbar spine, femoral neck, and total hip. Supplementary material 7

## CRediT authorship contribution statement

**David Castro Corredor:** Writing – review & editing, Writing – original draft, Visualization, Validation, Supervision, Project administration, Methodology, Investigation, Data curation, Conceptualization. **Luis Ángel Calvo Pascual:** Writing – review & editing, Writing – original draft, Validation, Software, Resources, Project administration, Methodology, Investigation, Formal analysis, Data curation.

## Informed consent

Written informed consent was obtained from all participants. Data were anonymised to protect confidentiality.

## Ethics approval

The protocol was approved by the central research ethics committee of Ciudad Real (study ID: 12/2023) in accordance with the Declaration of Helsinki.

## Funding

This study was supported by a research grant from the Spanish Society for Bone and Mineral Metabolism Research (SEIOMM) awarded during its 2024 annual congress in Seville.

## Declaration of competing interest

David Castro Corredor and Luis Ángel Calvo Pascual declare that they have no conflict of interest. Word count: 3100 (approx).

## Data Availability

Data will be made available on request.
